# SWATH2stats: An R/Bioconductor Package to Process and Convert Quantitative SWATH-MS Proteomics Data for Downstream Analysis Tools

**DOI:** 10.1371/journal.pone.0153160

**Published:** 2016-04-07

**Authors:** Peter Blattmann, Moritz Heusel, Ruedi Aebersold

**Affiliations:** 1 Department of Biology, Institute of Molecular Systems Biology, ETH Zurich, 8093, Zurich, Switzerland; 2 PhD program in Molecular and Translational Biomedicine, Competence Center Personalized Medicine UZH/ETH & Life Science Zurich Graduate School, ETH Zurich and University of Zurich, 8044, Zurich, Switzerland; 3 Faculty of Science, University of Zurich, 8057, Zurich, Switzerland; UGent / VIB, BELGIUM

## Abstract

SWATH-MS is an acquisition and analysis technique of targeted proteomics that enables measuring several thousand proteins with high reproducibility and accuracy across many samples. OpenSWATH is popular open-source software for peptide identification and quantification from SWATH-MS data. For downstream statistical and quantitative analysis there exist different tools such as MSstats, mapDIA and aLFQ. However, the transfer of data from OpenSWATH to the downstream statistical tools is currently technically challenging. Here we introduce the R/Bioconductor package SWATH2stats, which allows convenient processing of the data into a format directly readable by the downstream analysis tools. In addition, SWATH2stats allows annotation, analyzing the variation and the reproducibility of the measurements, FDR estimation, and advanced filtering before submitting the processed data to downstream tools. These functionalities are important to quickly analyze the quality of the SWATH-MS data. Hence, SWATH2stats is a new open-source tool that summarizes several practical functionalities for analyzing, processing, and converting SWATH-MS data and thus facilitates the efficient analysis of large-scale SWATH/DIA datasets.

## Introduction

**Fig 1:**
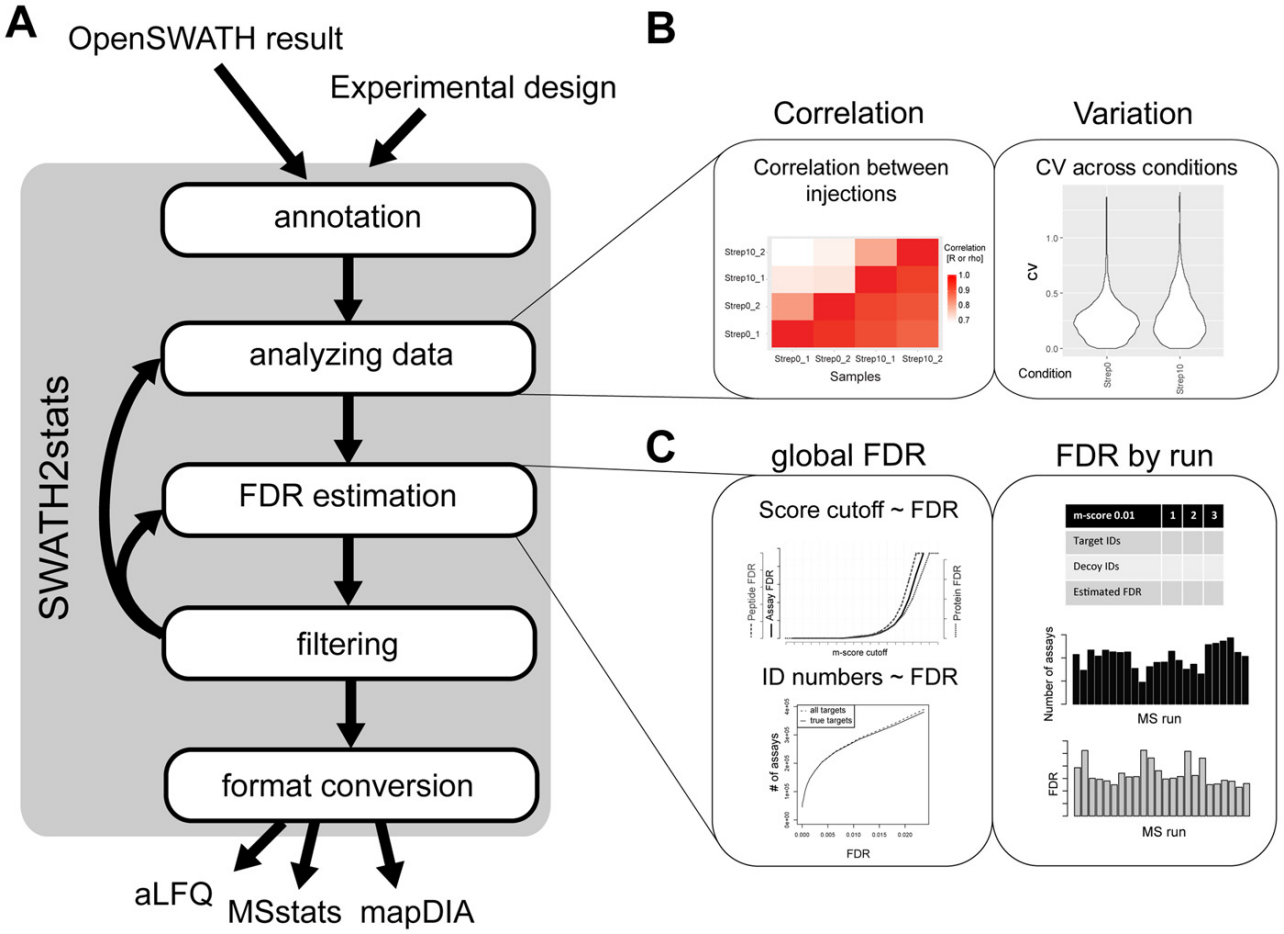
Overview of the R/Bioconductor package SWATH2stats. (A) SWATH2stats uses the OpenSWATH results or results from similar software. The information on the experimental design for annotation of the conditions and replicates can be provided separately or extracted from the OpenSWATH data. The data is processed along 5 different steps (annotation, analyzing the data, false discovery rate (FDR) estimation, filtering, format conversion) using different functions (Table 1) until the data can be directly exported in a suitable format for the downstream analysis tools aLFQ, MSstats, and mapDIA. (B) Shown are example plots from the package that show the correlation of signals between injections or the coefficient of variation (cv) across conditions. (C) Shown are example plots on how the estimated global FDR or FDR by run changes depending on different score criteria.

Targeted mass spectrometry-based proteomics allows the consistent and reproducible quantification of peptide analytes in complex samples [1]. SWATH-MS is a recently developed implementation of data-independent acquisition (DIA) and targeted analysis that increases the number of quantified peptides per sample compared to S/MRM by 2–3 orders of magnitude [2]. The SWATH-MS/DIA approach has become increasingly popular in proteomics. Different software tools have been developed for the identification and quantification of peptides from the highly convoluted fragment ion maps generated by DIA. These include OpenSWATH [3], a recent implementation of mProphet scoring in Skyline [4], DIA-Umpire [5], PeakView (ABSciex, Canada) and Spectronaut (Biognosys, Switzerland). Among these, the open-source OpenSWATH pipeline is a popular tool that produces a large tab-delimited results file containing the quantitative SWATH-MS data. The OpenSWATH pipeline consists of the OpenSWATH software [3] identifying and extracting quantitative data from targeted peptides within the fragment ion maps and a statistical assessment of the correct identification of these targeted peptides using the mProphet algorithm [6, 7]. For subsequent quantitative or statistical analyses of proteomic data, several tools have been developed by us and others: MSstats and mapDIA are tools that can be used to identify statistically significant differential expression of peptides and proteins in SWATH-MS data [8, 9]. The R package aLFQ allows absolute label free quantification of proteins in SWATH-MS data [10]. To interface the OpenSWATH output with these tools, the data needs to be processed into the respective input format, a task that can be challenging and time-consuming, due to the size of the data and programming skills required. Before subjecting it to further downstream statistical or quantitative analysis, the data typically needs to be annotated and an initial quality assessment performed. This step can also be used to filter for a subset of the data that will then be tested for differential expression. At the moment no tool exists to facilitate such different tasks for SWATH-MS data. Here we present a convenient R/Bioconductor package called SWATH2stats that allows to i) annotate the data, ii) analyze reproducibility across replicates, iii) estimate the FDR, iv) filter for assays meeting certain confidence or other criteria and v) convert the large proteomic datasets to the respective input formats of the downstream analysis tools MSstats, mapDIA, and aLFQ [8–10] (Fig 1A and Table 1).

**Table 1:** Functions included in the SWATH2stats package.

Category	Function names	Short description of function
**Load and annotation**	sample_annotation	Annotates the samples with the study design (Replicates, Conditions)
	reduce_OpenSWATH_output	Reduces the data table to fewer columns
	transform_MSstats_OpenSWATH	Converts the data from an MSstats like format to the OpenSWATH format
	import_data	Transforms the column names from a data frame to the required format for SWATH2stats
**Analyzing data**	count_analytes	Counts the analytes across the different samples
	plot_correlation_between_samples	Plots the correlation between samples
	plot_variation	Plots the coefficient of variation across samples
	plot_variation_vs_total	Plots the coefficient of variation in the whole data versus within replicates
	write_matrix_peptides	Calculates the summed signal per peptide in the different samples
	write_matrix_proteins	Calculates the summed signal per protein in the different samples
**FDR estimation**	assess_decoy_rate	Counts the number of decoy assays in the data
	assess_fdr_byrun	Estimates the FDR using a target decoy approach within each run
	assess_fdr_overall	Estimates the FDR using a target decoy approach across all runs
	mscore4assayfdr	Calculates an m-score threshold for reaching a certain assay FDR
	mscore4pepfdr	Calculates an m-score threshold for reaching a certain peptide FDR
	mscore4protfdr	Calculates an m-score threshold for reaching a certain protein FDR.
	plot.fdr_cube	Generates plots from fdr_cube objects (FDR estimates by run)
	plot.fdr_table	Generates plots from fdr_table objects (FDR estimates overall)
**Filtering**	filter_proteotypic_peptides	Selects only data from peptides that are proteotypic
	filter_mscore	Selects only data quantified with a certain m-score threshold
	filter_mscore_condition	Selects only data quantified with a certain m-score threshold and quantified a certain number of times within a condition
	filter_mscore_freqobs	Selects only data quantified with a given m-score threshold and a certain frequency of observation across the different samples
	filter_on_max_peptides	Selects only a given number of highest intense peptides per protein
	filter_on_min_peptides	Selects only proteins that have a minimal number of peptides quantified
	disaggregate	Transforms the data into transition-level format
**Format conversion**	convert4pythonscript	Converts the data into the format to be used by a supplied pythonscript to transform large data into transition-level format
	convert4aLFQ	Converts the data into the format for the R package aLFQ
	convert4mapDIA	Converts the data into the format for the C++ software mapDIA
	convert4MSstats	Converts the data into the format for the R package MSstats

## Material and Methods

### Implementation

SWATH2stats was programmed as an R package and is available on Bioconductor [11] (http://bioconductor.org/packages/SWATH2stats/). A vignette contained within the package explains the analysis procedure in detail. Dedicated explanation of each function is provided in the manual pages within the package. The functions can be grouped into five areas: i) Data loading and annotation, ii) analyzing the variation and correlation of the data, iii) FDR estimation, iv) data filtering and v) format conversion (see Fig 1A and Table 1). In addition to the base R functions, the package builds directly upon functions from the packages ggplot2 [12], reshape2 [13], data.table and grid.

### *S*.*pyogenes* dataset

In order to show the usage of SWATH2stats, an example script is presented (S1 and S2 Files). This script can be used to process a publicly available SWATH-MS dataset obtained from *S*.*pyogenes* [3]. This dataset contains four injections of *S*.*pyogenes* exposed to 0% or 10% human plasma with two biological replicates each. The SWATH-MS data was originally searched with the OpenSWATH pipeline using an assay library for *S*. *pyogenes* [3]. The results table used in the example script was obtained from PeptideAtlas (www.peptideatlas.org, PASS00289).

## Results

### Loading of SWATH data and annotation

The SWATH2stats package can process SWATH data from the integrated OpenSWATH pipeline (Rost et al. 2014, Teleman et al. 2015) (S1 File). Alternatively data from other proteomic software can be used when exported to a tab-delimited table where each row represents the quantitative results of one quantified targeted precursor peptide for each sample injection. The minimal information per row required is i) the assignment for each targeted peptide to a protein, ii) in which MS injection the peptide was quantified, and iii) a measure for the signal that was quantified. A score representing the confidence of identification needs to be present both for the target and decoy peptides in order to estimate an FDR with SWATH2stats (for the OpenSWATH results the m-score is used). In addition to the quantitative data, a table containing the meta-data for the experimental design can be provided in order to annotate the SWATH-MS results in SWATH2stats. This table needs to specify for each MS injection to which treatment condition it belongs and define the replicates of the same treatment condition. An example experimental design file is provided within the package and the example script shows how this information can be retrieved from the filename within the data if all the information is contained within the filename (S1 File).

### Visualization of data and variation between biological replicates

The SWATH2stats package provides different functions to directly analyze the results (Table 1). These functions provide functionality to count the number of analytes detected, or analyze the correlation and difference in their signal across the measured samples. A table with the summed signal per peptide or protein can be generated. Furthermore, the correlation and coefficient of variation for the signal between replicates and across all samples can be plotted (Fig 1B). These functions are useful to obtain a first impression of the data, but can also be used to assess the effect of filtering towards the signal or correlation across replicates.

### Estimation of the FDR on peptide and protein level

When analyzing many runs in parallel, false positive identifications can accumulate in the combined results table, resulting in a higher overall FDR than in one individual run. In addition, the FDR on peptide or protein level is typically higher than on the assay level [10, 14]. Therefore, it is important to control the peptide and protein FDR in large proteomics studies [15]. Here, we implemented an estimation of the FDR based on the target-decoy approach using a correction factor for the ratio of decoys to false targets (called fraction of false targets (FFT) or π_0_) [16–18]. The functions in this package support the estimation of the global FDR (Fig 1B) over multiple runs or within single runs (Fig 1C). These functions estimate the FDR on assay, peptide or protein level by counting the decoy assays, peptides or proteins passing a certain m-score criterion. In contrast to the naïve target decoy approach, the FDR estimate is corrected by the FFT or π_0_ [16–18]. All of these functions provide plots for visual inspection and can also be used to estimate a more stringent m-score/FDR criterion in order to reach a target FDR.

### Filtering the data

Depending on the aim of the downstream analysis, the data might need to be filtered further. For example, a more stringent score criterion can be set to only include data identified at a higher confidence. This can reduce the overall peptide or protein FDR of the data. For some projects, peptides that have not been identified reproducibly across a certain number of conditions should be excluded from further analysis. Therefore, another option is to filter for peptides that were identified across a certain number of injections or replicates. Another possibility is to select only proteins for which two independent peptides were quantified. Typically, these approaches lead to a preferential selection of true versus false targets or decoys and hence reduce the FDR in the data. Furthermore, filters are available to select the data for peptides present in only one protein (proteotypic peptides), or select n peptides per protein showing the highest signal (top n approach). In summary, SWATH2stats provides different functions that allow the user to filter the data based on i) meta-data from experimental design, ii) frequency of observation across samples, iii) number of sibling peptides (peptides mapping to the same protein entry) or on iv) m-score/FDR criteria (Table 1). Such filters can also be applied in combination, e.g. selecting proteotypic peptides that have been quantified in more than 50% of the samples with an estimated FDR on assay level lower than 0.001. The filters are equally applied to the decoy assays and thus the effect of these filters on false targets can be estimated by re-assessing the decoy-estimated FDR. The application of the FDR estimation functions in interplay with the filtering functions can help the researcher in selecting an efficient strategy to establish the highest possible data quality.

### Conversion of the data

In the last step SWATH2stats offers functions to convert the OpenSWATH data to a format required for popular statistical tools such as the R/Bioconductor package MSstats [8], the C++ tool mapDIA [9], as well as the quantitative proteomics R package aLFQ [10] (Fig 1A and Table 1). During this conversion, the data table changes from a peakgroup-level format (one row per peakgroup) to a transition-level format (one row per transition), or from a long format to a wide format (the signal for different samples is stored in a single column to a format where the signal for each sample is present in different columns) (Table 1). The converted data can then directly be read by the different downstream statistical or quantitative tools.

## Discussion

The R/Bioconductor package SWATH2stats establishes for the first time a convenient link between the OpenSWATH pipeline [3, 7] and different downstream analysis tools such as MSstats [8] and mapDIA [9]. In addition, it enables annotation, analysis, FDR estimation, and filtering of the data with different functions (Table 1). The SWATH2stats package thus enables efficient and convenient data quality control and visualization that helps to improve the quality of the subsequent statistical and quantitative results. The SWATH2stats package has been documented with a detailed vignette and deposited on the popular R/Bioconductor platform. The implementation within R allows the direct usage of other plotting and statistical functions and the open-source implementation allows full transparency on how the data is processed. SWATH2stats is specifically targeted for SWATH projects with samples from many different treatments and containing biological replicates. The implementation of SWATH2stats in the popular framework of R/Bioconductor [11] and its ease-of-use is expected to significantly facilitate end-to-end analysis of large-scale SWATH/DIA datasets for users.

## Supporting Information

S1 File SWATH2stats example script. Example R code showing the usage of the SWATH2stats package. The data processed is the publicly available dataset of S.pyogenes (Röst et al. 2014, www.peptideatlas.org; PASS 00289) and was processed with SWATH2stats v 1.1.14.
(PDF)

S2 File R markdown source file for SWATH2stats example script. R markdown file that was used to generate the S1 File.
(RMD)
