# Supplementary material for: SWATH2stats: An R/Bioconductor Package to Process and Convert Quantitative SWATH-MS Proteomics Data for Downstream Analysis Tools
Source: PLoS One. 2016 Apr 7;11(4):e0153160. doi: 10.1371/journal.pone.0153160 (PMC4824525; doi:10.1371/journal.pone.0153160)
Supplement: S1 File — Example R code showing the usage of the SWATH2stats package. The data processed is the publicly available dataset of S.pyogenes (Röst et al. 2014, www.peptideatlas.org; PASS 00289) and was processed with SWATH2stats v 1.1.14. (PDF) [file pone.0153160.s001.pdf]

# SWATH2stats example script

Example R code showing the usage of the SWATH2stats package. The data processed is the publicly available dataset of *S.pyogenes* (Röst et al. 2014) (http://www.peptideatlas.org/PASS/PASS00289). The results file 'rawOpenSwathResults_1pcnt_only.tsv' can be found on PeptideAtlas (ftp://PASS00289@ftp.peptideatlas.org/../Spyogenes/results/). This is a R Markdown file, showing the result of processing this data. The lines shaded in grey represent the R code executed during this analysis.

The stable release package SWATH2stats can be directly installed from Bioconductor using the commands below. This file here was generated using the current development release SWATH2stats v.1.1.14 that can be downloaded from http://bioconductor.org/packages/devel/bioc/html/SWATH2stats.html.

```
## try http:// if https:// URLs are not supported
source('https://bioconductor.org/biocLite.R')
biocLite('SWATH2stats')
```

## Part 1: Loading and annotation

Load the SWATH-MS example data from the package, this is a reduced file in order to limit the file size of the package.

```
library(SWATH2stats)
library(data.table)
data('Spyogenes', package = 'SWATH2stats')
```

Alternatively the original file downloaded from the Peptide Atlas can be loaded from the working directory.

```
data <- data.frame(fread('rawOpenSwathResults_1pcnt_only.tsv', sep='\t', header=TRUE))
```

Extract the study design information from the file names. Alternatively, the study design table can be provided as an external table.

```
Study_design <- data.frame(Filename = unique(data$align_origfilename))
Study_design$Filename <- gsub(".*strep_align/(.*)_all_peakgroups.*", "\\1",
    Study_design$Filename)
Study_design$Condition <- gsub("(Strep.*)_Repl.*", "\\1", Study_design$Filename)
Study_design$BioReplicate <- gsub(".*Repl([[:digit:]])_.*", "\\1", Study_design$Filename)
Study_design$Run <- seq(1:nrow(Study_design))
head(Study_design)
```

```
##                                  Filename Condition BioReplicate Run
## 1  Strep0_Repl1_R02/split_hroest_K120808    Strep0            1   1
## 2  Strep0_Repl2_R02/split_hroest_K120808    Strep0            2   2
## 3 Strep10_Repl1_R02/split_hroest_K120808   Strep10            1   3
## 4 Strep10_Repl2_R02/split_hroest_K120808   Strep10            2   4
```

The SWATH-MS data is annotated using the study design table.

```r
data.annotated <- sample_annotation(data, Study_design)
```

Remove the decoy peptides for a subsequent inspection of the data.

```r
data.annotated.nodecoy <- subset(data.annotated, decoy==FALSE)
```

## Part 2: Analyze correlation, variation and signal

Count the different analytes for the different injections.

```
count_analytes(data.annotated.nodecoy)
```

```
##        run_id transition_group_id FullPeptideName ProteinName
## 1  Strep0_1_1               10229            8377        1031
## 2  Strep0_2_2                9716            7970        1003
## 3 Strep10_1_3                8692            7138         943
## 4 Strep10_2_4                8424            6941         910
```

Plot the correlation of the signal intensity.

```
correlation <- plot_correlation_between_samples(data.annotated.nodecoy, column.values = 'Intensity')
```

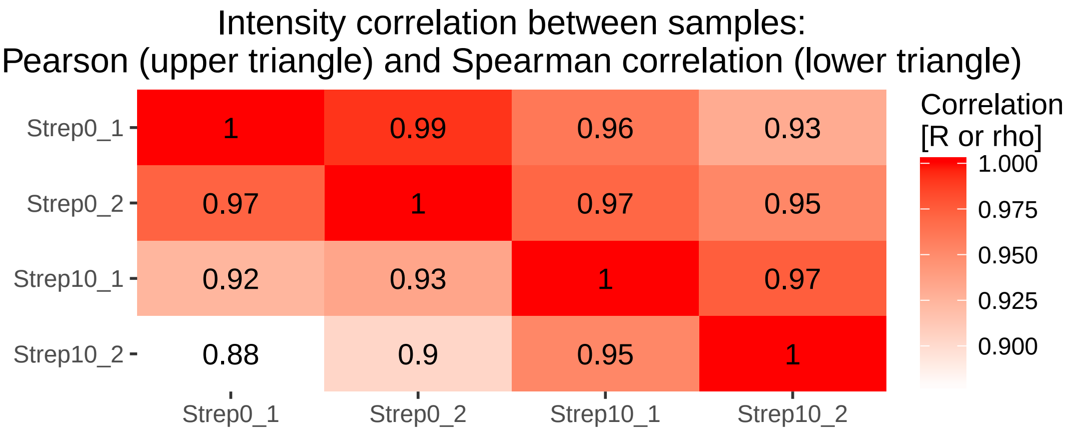


Plot the correlation of the delta_rt, which is the deviation of the retention time from the expected retention time.

```
correlation <- plot_correlation_between_samples(data.annotated.nodecoy, column.values = 'delta_rt')
```

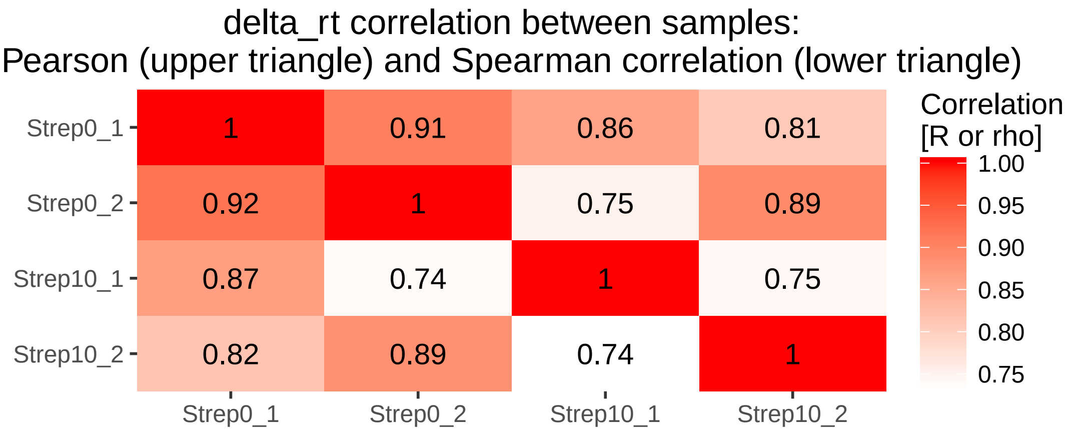

Plot the variation of the signal across replicates.

```r
variation <- plot_variation(data.annotated.nodecoy)
```

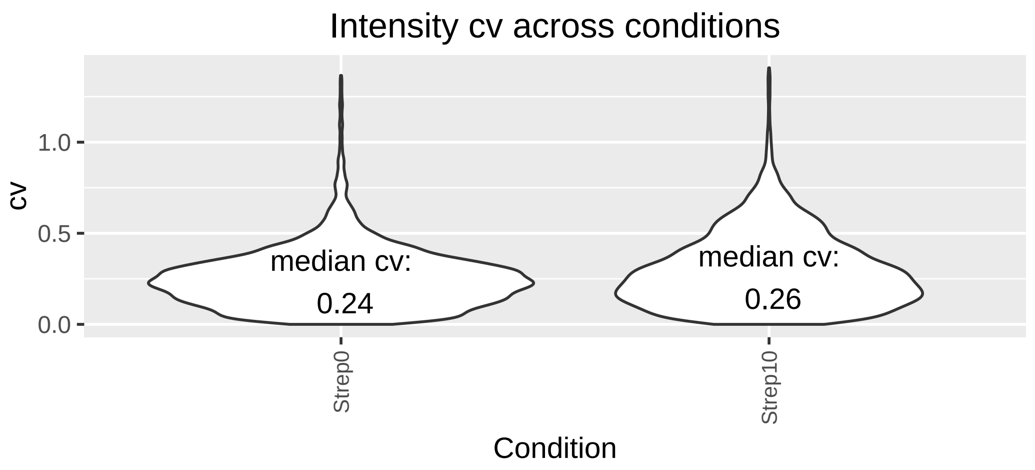


```r
variation[[2]]
```

```
##   Condition   mode_cv   mean_cv median_cv
## 1    Strep0 0.2280372 0.2545450 0.2351859
## 2   Strep10 0.1706934 0.2947144 0.2592725
```

Plot the total variation versus variation within replicates.

```r
variation_total <- plot_variation_vs_total(data.annotated.nodecoy)
```

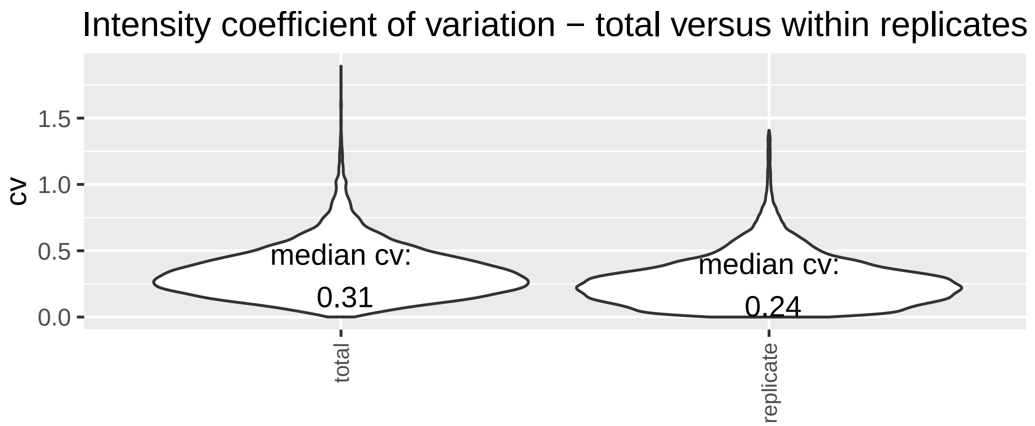


```r
variation_total[[2]]
```

```
##       scope   mode_cv   mean_cv median_cv
## 1 replicate 0.2209867 0.2728681 0.2438041
## 2     total 0.2655678 0.3439050 0.3139993
```

Calculate the summed signal per peptide and protein across samples.

```r
peptide_signal <- write_matrix_peptides(data.annotated.nodecoy)
protein_signal <- write_matrix_proteins(data.annotated.nodecoy)
head(protein_signal)
```

```
##                        ProteinName Strep0_1_1 Strep0_2_2 Strep10_1_3
## 1  Spyo_Exp3652_DDB_SeqID_1571119     265206     163326       51831
## 2  Spyo_Exp3652_DDB_SeqID_1579753     185725     150672       21483
## 3  Spyo_Exp3652_DDB_SeqID_1631459     176686     132415       42165
## 4  Spyo_Exp3652_DDB_SeqID_1640263       3310       6617       98550
## 5  Spyo_Exp3652_DDB_SeqID_1709452     852502     747772      503581
## 6 Spyo_Exp3652_DDB_SeqID_17244480      17506      29578        7607
##   Strep10_2_4
## 1       45021
## 2      144314
## 3       32735
## 4       45169
## 5      504761
## 6        2482
```

## Part 3: FDR estimation

Estimate the overall FDR across runs using a target decoy strategy.

```
par(mfrow = c(1, 3))
fdr_target_decoy <- assess_fdr_overall(data.annotated, n.range = 10, FFT = 0.25, output = 'Rconsole')
```

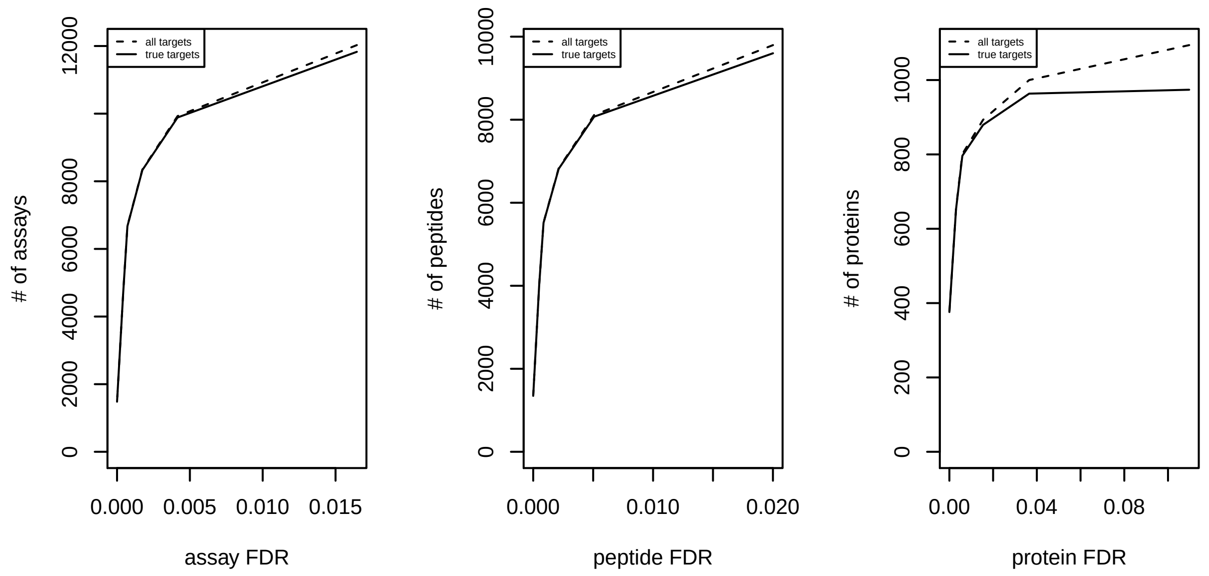


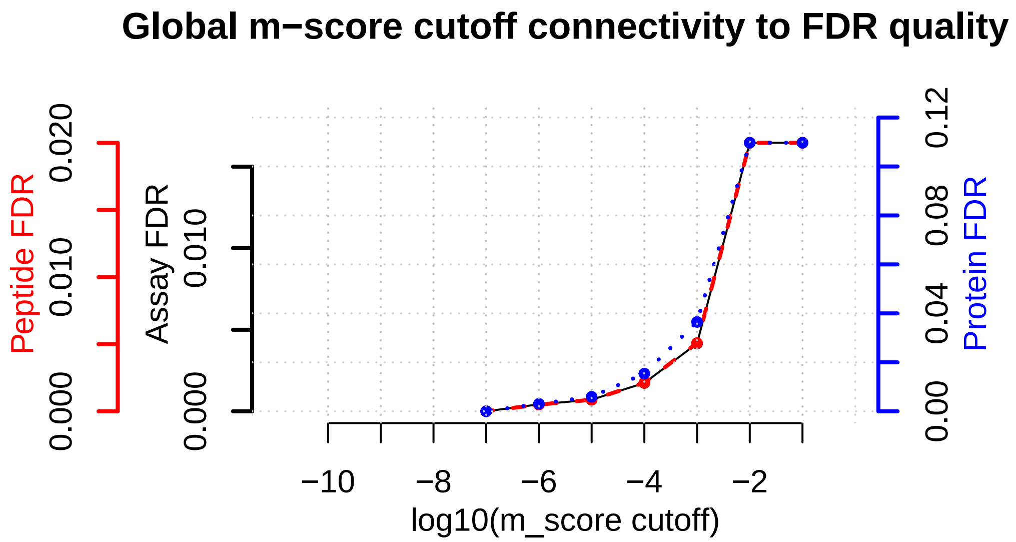


According to this FDR estimation one would need to filter the data with a lower mscore threshold to reach an overall protein FDR of 5%.

```r
mscore4protfdr(data, FFT = 0.25, fdr_target = 0.05)
```

```
## Target protein FDR:0.05
```

```
## Required overall m-score cutoff:0.0017783
## achieving protein FDR =0.0488
```

```
## [1] 0.001778279
```

# Part 4: Filtering

Filter data for values that pass the 0.001 mscore criteria in at least two replicates of one condition.

```r
data.filtered <- filter_mscore_condition(data.annotated, 0.001, n.replica = 2)
```

```
## Fraction of peptides selected: 0.67
```

```
## Dimension difference: 7226, 0
```

Select only the 10 peptides showing strongest signal per protein.

```r
data.filtered2 <- filter_on_max_peptides(data.filtered, n_peptides = 10)
```

```
## Before filtering:
##   Number of proteins: 884
##   Number of peptides: 6594
##
## Percentage of peptides removed: 29.6%
##
## After filtering:
##   Number of proteins: 884
##   Number of peptides: 4642
```

Filter for proteins that are supported by at least two peptides.

```
data.filtered3 <- filter_on_min_peptides(data.filtered2, n_peptides = 2)
```

```
## Before filtering:
##   Number of proteins: 884
##   Number of peptides: 4642
##
## Percentage of peptides removed: 3.6%
##
## After filtering:
##   Number of proteins: 717
##   Number of peptides: 4475
```

# Part 5: Conversion

Convert the data into a transition-level format (one row per transition measured).

```
data.transition <- disaggregate(data.filtered3)
```

```
## The library contains 6 transitions per precursor.
##
## The data table was transformed into a table containing one row per transition.
```

Convert the data into the format required by MSstats.

```
MSstats.input <- convert4MSstats(data.transition)
```

```
## One or several columns required by MSstats were not in the data.
## Missing columns: ProductCharge, IsotopeLabelType
```

```
## IsotopeLabelType was filled with light.
```

```
## Warning in convert4MSstats(data.transition): Intensity values that were 0,
## were replaced by NA
```

```
head(MSstats.input)
```

```
##                        ProteinName    PeptideSequence PrecursorCharge
## 1 Spyo_Exp3652_DDB_SeqID_1571119 AEAAIYQFLEAIGENPNR               3
## 2 Spyo_Exp3652_DDB_SeqID_1571119 AEAAIYQFLEAIGENPNR               3
## 3 Spyo_Exp3652_DDB_SeqID_1571119 AEAAIYQFLEAIGENPNR               3
## 4 Spyo_Exp3652_DDB_SeqID_1571119 AEAAIYQFLEAIGENPNR               3
## 5 Spyo_Exp3652_DDB_SeqID_1571119        AHIAYLPSDGR               2
## 6 Spyo_Exp3652_DDB_SeqID_1571119        AHIAYLPSDGR               2
##                        FragmentIon ProductCharge IsotopeLabelType Intensity
## 1 105801_AEAAIYQFLEAIGENPNR/3_y6            NA            light      4752
## 2 105801_AEAAIYQFLEAIGENPNR/3_y6            NA            light      6144
## 3 105801_AEAAIYQFLEAIGENPNR/3_y6            NA            light      3722
```

```
## 4 105801_AEAAIYQFLEAIGENPNR/3_y6            NA            light      6624
## 5        118149_AHIAYLPSDGR/2_y8            NA            light      4036
## 6        118149_AHIAYLPSDGR/2_y8            NA            light      1642
##   BioReplicate Condition Run
## 1            2    Strep0   2
## 2            1   Strep10   3
## 3            2   Strep10   4
## 4            1    Strep0   1
## 5            1    Strep0   1
## 6            1   Strep10   3
```

Convert the data into the format required by mapDIA.

```
mapDIA.input <- convert4mapDIA(data.transition)
head(mapDIA.input)
```

```
##                      ProteinName      PeptideSequence
## 1 Spyo_Exp3652_DDB_SeqID_1571119   AEAAIYQFLEAIGENPNR
## 2 Spyo_Exp3652_DDB_SeqID_1571119          AHIAYLPSDGR
## 3 Spyo_Exp3652_DDB_SeqID_1571119             EEFTAVFK
## 4 Spyo_Exp3652_DDB_SeqID_1571119 EKAEAAIYQFLEAIGENPNR
## 5 Spyo_Exp3652_DDB_SeqID_1571119            EQHEDVVIVK
## 6 Spyo_Exp3652_DDB_SeqID_1571119     LTSQIADALVEALNPK
##                      FragmentIon Strep0_1 Strep0_2 Strep10_1 Strep10_2
## 1  105801_AEAAIYQFLEAIGENPNR/3_y6     6624     4752      6144      3722
## 2         118149_AHIAYLPSDGR/2_y8     4036     2405      1642       720
## 3             35179_EEFTAVFK/2_y5     2307     1541      1561       NaN
## 4 28903_EKAEAAIYQFLEAIGENPNR/3_y6     3410     2185       NaN      1984
## 5           73581_EQHEDVVIVK/2_b6     2423     1343       NaN       NaN
## 6   115497_LTSQIADALVEALNPK/2_y11     6553     6349       NaN       NaN
```

Convert the data into the format required by aLFQ.

```
aLFQ.input <- convert4aLFQ(data.transition)
head(aLFQ.input)
```

```
##       run_id                     protein_id         peptide_id
## 1  Strep0_2_2 Spyo_Exp3652_DDB_SeqID_1571119 AEAAIYQFLEAIGENPNR
## 2 Strep10_1_3 Spyo_Exp3652_DDB_SeqID_1571119 AEAAIYQFLEAIGENPNR
## 3 Strep10_2_4 Spyo_Exp3652_DDB_SeqID_1571119 AEAAIYQFLEAIGENPNR
## 4  Strep0_1_1 Spyo_Exp3652_DDB_SeqID_1571119 AEAAIYQFLEAIGENPNR
## 5  Strep0_1_1 Spyo_Exp3652_DDB_SeqID_1571119        AHIAYLPSDGR
## 6 Strep10_1_3 Spyo_Exp3652_DDB_SeqID_1571119        AHIAYLPSDGR
##                                      transition_id   peptide_sequence
## 1 AEAAIYQFLEAIGENPNR 105801_AEAAIYQFLEAIGENPNR/3_y6 AEAAIYQFLEAIGENPNR
## 2 AEAAIYQFLEAIGENPNR 105801_AEAAIYQFLEAIGENPNR/3_y6 AEAAIYQFLEAIGENPNR
## 3 AEAAIYQFLEAIGENPNR 105801_AEAAIYQFLEAIGENPNR/3_y6 AEAAIYQFLEAIGENPNR
## 4 AEAAIYQFLEAIGENPNR 105801_AEAAIYQFLEAIGENPNR/3_y6 AEAAIYQFLEAIGENPNR
## 5              AHIAYLPSDGR 118149_AHIAYLPSDGR/2_y8        AHIAYLPSDGR
## 6              AHIAYLPSDGR 118149_AHIAYLPSDGR/2_y8        AHIAYLPSDGR
##   precursor_charge transition_intensity concentration
## 1                3                 4752             ?
```

```
## 2                 3                 6144             ?
## 3                 3                 3722             ?
## 4                 3                 6624             ?
## 5                 2                 4036             ?
## 6                 2                 1642             ?
```

Session info on the R version and packages used.

```r
sessionInfo()
```

```
## R version 3.2.3 (2015-12-10)
## Platform: x86_64-w64-mingw32/x64 (64-bit)
## Running under: Windows 7 x64 (build 7601) Service Pack 1
##
## locale:
## [1] LC_COLLATE=German_Switzerland.1252  LC_CTYPE=German_Switzerland.1252
## [3] LC_MONETARY=German_Switzerland.1252 LC_NUMERIC=C
## [5] LC_TIME=German_Switzerland.1252
##
## attached base packages:
## [1] stats     graphics  grDevices utils     datasets  methods   base
##
## other attached packages:
## [1] data.table_1.9.6   SWATH2stats_1.1.14
##
## loaded via a namespace (and not attached):
##  [1] Rcpp_0.12.3      digest_0.6.9     chron_2.3-47     grid_3.2.3
##  [5] plyr_1.8.3       gtable_0.2.0     formatR_1.2.1    magrittr_1.5
##  [9] scales_0.4.0     evaluate_0.8     ggplot2_2.1.0    stringi_1.0-1
## [13] reshape2_1.4.1   rmarkdown_0.9.5  labeling_0.3     tools_3.2.3
## [17] stringr_1.0.0    munsell_0.4.3    yaml_2.1.13      colorspace_1.2-6
## [21] htmltools_0.3    knitr_1.12.3
```
